# Genome Sequence of Morganella morganii DG56-16, Isolated from Shinisaurus crocodilurus

**DOI:** 10.1128/MRA.01301-18

**Published:** 2019-01-24

**Authors:** Hai-Ying Jiang, Li-Bo Lin, Ming-Wei Huang, Ying-An Zang, Jin-Ping Chen

**Affiliations:** a Guangdong Key Laboratory of Animal Conservation and Resource Utilization, Guangdong Public Laboratory of Wild Animal Conservation and Utilization, Guangdong Institute of Applied Biological Resources, Guangzhou, China; b Zhongkai University of Agriculture and Engineering, Guangzhou, China; University of Delaware

## Abstract

The complete genome sequence of Morganella morganii DG56-16 was sequenced. This strain was isolated from the liver of a dead crocodile lizard (Shinisaurus crocodilurus). The genome size was 3.9 Mb, with a G+C content of 50.9%.

## Abstract (precis)

The complete genome sequence of Morganella morganii DG56-16 was sequenced. This strain was isolated from the liver of a dead crocodile lizard (Shinisaurus crocodilurus).

## ANNOUNCEMENT

Morganella morganii is a Gram-negative bacterium belonging to the Enterobacteriaceae. It can be found in vertebrate intestines as a normal commensal and is widely distributed in the environment. In humans, this bacterium is a nonnegligent opportunistic pathogen that causes sepsis, abscess, cellulitis, etc. (1). In addition, M. morganii is an important pathogen of iatrogenic infection, with a high mortality rate (2, 3). Domestic and wildlife animal diseases and deaths caused by M. morganii also have been widely reported (4–6). Recently, more and more concerns have been concentrated on M. morganii because of its increased levels of antibiotic resistance and virulence (1, 4, 6). To date, 44 draft or complete genome sequences have been reported in GenBank. Here, we report a complete genome sequence of M. morganii DG56-16, isolated from the liver of a juvenile crocodile lizard that died from disease. This would benefit the understanding of the pathogenicity of M. morganii.

The liver was resected from the dead crocodile lizard, homogenized, and spread on a Columbia blood agar base plate (Oxoid Ltd., UK). The plate was incubated at 30°C for 24 h. Morganella morganii DG56-16 was isolated and purified by the streak plate method. Total DNA was extracted using the TIANamp bacteria DNA kit DP302 (Tiangen Biotech [Beijing] Co., Ltd., China). A SMRTbell library was constructed by SMRTbell template prep kit 1.0 (Pacific Biosciences of California, Inc., USA). Sequencing was performed using a PacBio system. Quality control of raw sequences and genome assembly were conducted by SMRT Link version 5.0.1.

Paired-end (150-bp) Illumina sequencing was performed to correct the assembled sequence that resulted from the PacBio system. The Illumina sequencing library was constructed using the TruSeq DNA PCR-free sample preparation kit (Illumina, Inc., USA). Clean data resulting from Illumina sequencing was mapped onto the PacBio assembly genome sequence using the BWA software (http://bio-bwa.sourceforge.net/bwa.shtml). Alignment duplications were removed using Samtools (http://www.htslib.org/). Mapping quality was filtered by a personal Perl script. The correction was performed using the filtered result.

Gene prediction was performed using GeneMarkS 4.28 (7). Interspersed repetitive sequences were predicted by RepeatMasker 4.0.7 (http://www.repeatmasker.org/). Tandem repeats were searched by TRF 4.09 (http://tandem.bu.edu/trf/trf.download.html). The tRNA genes were predicted by tRNAscan-SE 2.0 (8), and the rRNA genes were analyzed using RNAmmer 1.2 (9). The small nuclear RNAs were predicted by BLAST against the Rfam database (10). Genomic islands (GIs) were predicted by IslandPath-DIMOB 1.0.0 (11). The prophage prediction was conducted by PHAST (12). Clustered regularly interspaced short palindromic repeat (CRISPR) sequences were identified by CRISPRFinder. For gene function, DIAMOND version 0.9.16 (13), with an E value of ≤1e−5, was used for alignment against databases, and the item with highest score was selected. The databases included GO, KEGG, COG (14), NR, Pfam, TCDB (15), and Swiss-Prot. Default parameters were used in all softwares.

The total number of PacBio reads was 179,195, with an *N*_50_ read length of 9,194 bp. A total of 1,633 Mb was obtained by Illumina sequencing. Finally, the genome of M. morganii DG56-16 was assembled into a circular chromosome (3,902,034 bp), with 50.9% G+C content. The genome coverage of PacBio was ∼368×. The annotation results showed that 3,817 coding genes and 124 noncoding RNAs were included. In addition, 11 GIs, 5 prophage sequences, 147 interspersed repeat sequences, and 125 tandem repeat sequences were found. However, no CRISPRs were found.

### Data availability.

The raw and assembled sequences of M. morganii DG56-16 have been deposited in GenBank under accession number CP032295, BioProject number PRJNA490253, and SRA number SRP162219.
